# Maternal Pre-Pregnancy Obesity and Gestational Diabetes Mellitus Increase the Risk of Childhood Obesity

**DOI:** 10.3390/children9070928

**Published:** 2022-06-21

**Authors:** Mi Jin Choi, Juyoun Yu, Jimi Choi

**Affiliations:** 1 Department of Nursing, Changwon National University, Changwon-si 51140, Korea; mj061079@gmail.com; 2 Division of Endocrinology and Metabolism, Department of Internal Medicine, Korea University College of Medicine, Seoul 02841, Korea; jjimchoi@gmail.com

**Keywords:** gestational diabetes, maternal obesity, pediatric obesity

## Abstract

Previous studies have shown inconsistent results regarding the effects of maternal gestational diabetes mellitus (GDM) and pre-pregnancy obesity (PPO) on childhood obesity. This study aimed to determine the risk for early childhood obesity based on maternal GDM and PPO. This nationwide study used data obtained from the National Health Information Database in South Korea. The participants were divided into four groups based on maternal GDM and PPO, and 1:1 matching was performed. Each group had 1319 participants. A generalized estimating equation model was used to analyze the changes in body mass index percentile of children with age, and simple and multiple conditional logistic regression models were used to compare the prevalence of childhood obesity at 5 years. Children whose mothers had both PPO and GDM, only PPO, or only GDM had a 4.46 (95% CI: 3.28–6.05, *p* < 0.001), 3.11 (95% CI: 2.27–4.26, *p* < 0.001), or 1.58 (95% CI: 1.12–2.23, *p* = 0.010) times higher risk, respectively, of developing childhood obesity than children whose mothers had neither PPO nor GDM. Maternal PPO increases the risk for childhood obesity to a higher degree than maternal GDM, and the presence of both increases the risk even further.

## 1. Introduction

Childhood obesity is a serious global health issue [1]. It continues beyond school age and adolescence and progresses to adult obesity, increasing the risk for metabolic conditions, such as diabetes mellitus and cardiovascular diseases [2,3,4]. Early childhood constitutes the basis for physical growth and later impacts adult lifestyle choices. Thus, given its long-lasting effects and adaptability, early childhood is regarded as the most important period to implement preventive measures against obesity [5,6]. In 2020, the World Health Organization (WHO) estimated that 38.9 million children between the ages of 0 and 5 years were either overweight or obese (with a prevalence rate of 5.7%) [1]. To emphasize the importance of early childhood obesity management, the WHO established the Commission on Ending Childhood Obesity in 2020 with the aim of decreasing the prevalence of obesity among children below the age of 5 years [7].

Childhood obesity is a multidimensional, complex disease, and factors such as genetics, physiological, environmental, and behavioral factors of the child, family, and society have a large impact. Importantly, the risk of childhood obesity begins before birth [8,9], and thus its effective prevention requires early intervention based on understanding its prenatal origin [10]. The most common prenatal causes of childhood obesity include maternal gestational diabetes mellitus (GDM) and pre-pregnancy obesity (PPO) [11]. Baker and Osmond’s fetal programming concept illustrates that the nutritional supply from pregnant women and the intrauterine environment affects the development and function of fetal tissue [12]. Particularly, children of women with high blood glucose levels become overweight and obese during infancy and childhood and are at an increased risk for metabolic diseases such as obesity and diabetes mellitus [13,14,15].

Although several studies on the long-term effects of maternal high blood glucose levels during pregnancy on childhood have been conducted, starting from the popular early Pima Indian study [16,17], the findings have been conflicting. As a result, the association between GDM and childhood obesity is not yet clearly identified [18,19]. The inconsistent results may be attributed to differences in the characteristics of study participants that were associated with obesity risk, the age of children at the time of obesity assessment, the types of GDM or diagnostic criteria used to identify GDM, having different study designs with respect to control groups, or different ways of controlling for confounding variables [18,19]. Furthermore, the effects of maternal PPO and birth weight on the association between GDM and risk for childhood obesity remain controversial [20,21]. PPO is a risk factor for both GDM and childhood obesity [22], but it is difficult to identify the individual impact of these factors. Thus, the association between PPO, GDM, and childhood obesity remains unclear.

Prevention strategies for the prospect of childhood obesity may change depending on the presence of PPO and GDM, and thus, the influence of these factors must be clearly identified first. Therefore, this study aimed to determine the risk for early childhood obesity (at 3, 4, and 5 years of age) by examining the effects of maternal PPO and GDM separately.

## 2. Materials and Methods

### 2.1. Data Source

The National Health Insurance Service (NHIS) administers the compulsory health insurance scheme that covers the entire population of South Korea and provides data through the National Health Information Database (NHID), which is linked to multiple national secondary databases maintained by other government agencies [23]. This nationwide study used data from the NHIS-NHID and included information on medical service usage from the healthcare utilization databases, behavioral health information from the health screening database, and sociodemographic information from the eligibility database. Data for children were obtained from the infant health screening database, which includes the results of mandatory childhood examinations for growth, developmental assessments, dental examinations, and infant healthcare education since 2008. As part of these examinations, infants and children receive 7 health check-ups at 4–6, 9–12, 18–24, 30–36, 42–48, 54–60, and 66–71 months of age. This study was approved by both the institutional review board of Chodang University (approval number: CIRB-2021-07-02; approval date: 7 July 2021) and the NHIS review board (NHIS-2021-1-643), and the requirement for informed consent was waived because of the retrospective study design. The NHID data were anonymized such that the researchers did not have access to personal identifiers for included mother–child pairs. Confidentiality was strictly maintained during all study periods in accordance with the relevant regulations of the NHIS.

### 2.2. Study Participants and Covariates

The study participants were children born between 2009 and 2012 who underwent both the fourth (30–36 months) and sixth (54–60 months) screenings of the infant health check-up. The fourth and sixth screenings for infants were the data points of this study. The health insurance number for mother-child and the year of mother’s delivery and child’s birth were used to confirm the links of study participants with their mothers. Multifetal pregnancies and premature babies with gestational age under 37 weeks were excluded.

**Figure 1:**
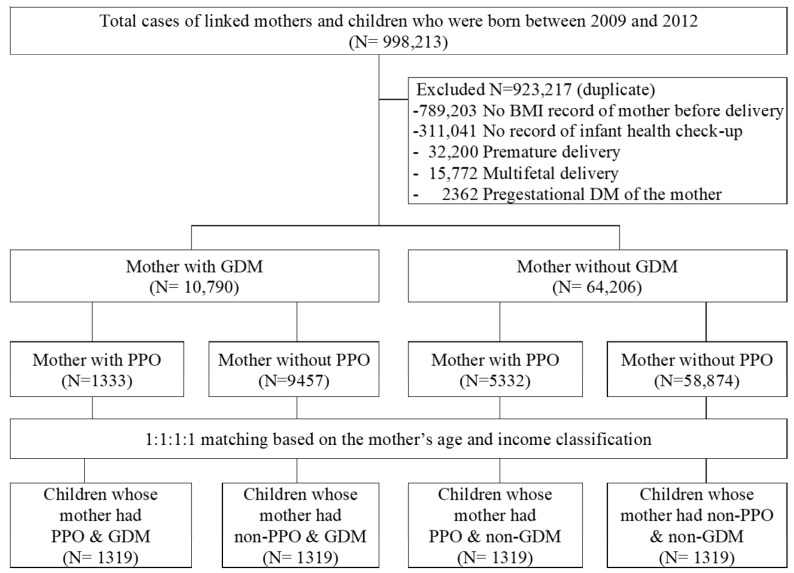
Diagrammatic representation of selection and classification of participants. BMI: body mass index, DM: diabetes mellitus, GDM: gestational diabetes mellitus, PPO: pre-pregnancy obesity.

The participants were divided into four groups based on maternal GDM and PPO to determine their influence on obesity incidence in the children. Maternal GDM was identified according to the GDM code of the International Classification of Diseases (O24.4) during the second and third trimesters of pregnancy [24]. Children whose mothers had pre-existing diabetes (code E10-E14 or O24) before and during the first trimester of pregnancy or unspecified gestational diabetes (code O24.0–O24.3, 24.9) were excluded. Mothers who had never been diagnosed with any type of DM before and during any pregnancy were considered mothers without GDM. Maternal PPO was defined as having a body mass index (BMI) >25 on a health screening within 2 years from 280 days before delivery. The four study groups were GDM with PPO, GDM without PPO, non-GDM with PPO, and non-GDM without PPO. To set up comparisons, 1:1 matching was performed by selecting the year of birth, mother’s age, and income level (low, middle, or high according to the insurance fee decile) as matching variables. As a result, there were 1319 study participants in each group, as shown in Figure 1.

Children’s BMI percentile, sex, birth weight, breastfeeding, diet, television (TV) or screen time, and preference for exercise were retrieved from the infant health screening database. The BMI percentile, which was part of the fourth, fifth, and sixth assessments, was used to examine the prevalence rate of childhood obesity among children aged 3–5 years old. According to the 2007 Korean pediatric growth chart, childhood obesity was defined as the 95th percentile above the BMI growth curve for different ages and sexes [25]. Sex and birth weight information were retrieved from the fourth assessment, and details about breastfeeding were retrieved from the data from the first assessment when available. Data on the consumption of sweetened drinks and fast food were obtained from the fourth and sixth assessments, respectively. Viewing TV or screen time and the preference for exercise were acquired from the sixth assessment data.

### 2.3. Data Analysis

Data are presented as frequency (percentage) for categorical variables and mean (standard deviation) for continuous variables. We used the generalized estimating equation (GEE) method with an appropriate link function to compare the baseline characteristics among groups while accounting for the correlation within matched participants. To analyze the changes in the BMI percentile of children with age, we used a GEE model assuming a normal distribution with an identity link function and included an interaction term between age and BMI percentile. Simple and multiple conditional logistic regression models were used to compare the prevalence of obesity at 5 years. The covariates included in the multiple models were all related to characteristics of children, such as birth weight, sex, breastfeeding method, diet habits, TV or screen time, and exercise preference. The risk for childhood obesity in mothers with PPO and/or GDM compared to mothers without PPO and/or GDM was represented using odds ratios and corresponding 95% confidence intervals (CIs). A *p*-value < 0.05 was considered statistically significant, and all data analyses were performed using SAS Enterprise Guide version 7.1 (SAS Institute Inc., Cary, NC, USA).

## 3. Results

The characteristics of the four groups classified based on the presence of PPO and GDM among pregnant women are shown in Table 1. The average maternal BMI was 27.6 kg/m^2^ and 27.3 kg/m^2^ for the PPO groups with and without GDM and 20.7 kg/m^2^ and 20.6 kg/m^2^ for the non-PPO groups with and without GDM, respectively. Thus, there was a significant difference between the groups depending on the presence of PPO (*p* < 0.001), but there was no difference between the groups depending on the presence of GDM. Looking at the children’s characteristics in each group, children whose mothers had PPO showed a higher birth weight than those whose mothers did not have PPO (*p* < 0.001). When mothers had both PPO and GDM, the percentage of children who consumed more fast food and/or watched TV or screens for more than 2 h/day was significantly greater than for the other groups. There was no difference in sex, consumption of sweetened drinks, or exercise preference in the children across the groups.

**Figure 2:**
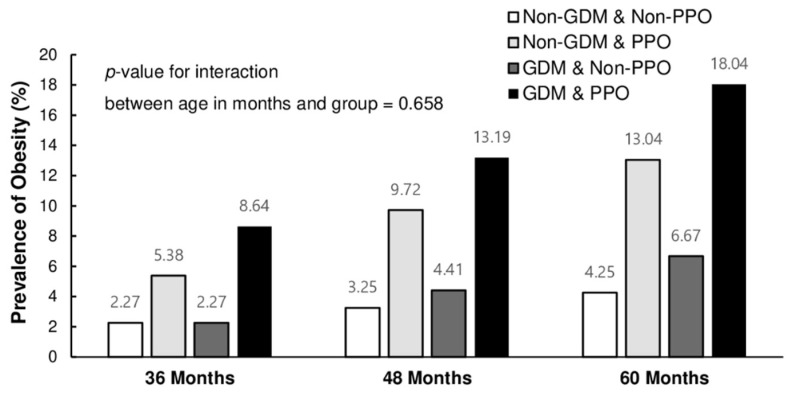
Prevalence of childhood obesity according to age and group. PPO: pre-pregnancy obesity, GDM: gestational diabetes mellitus.

**Figure 3:**
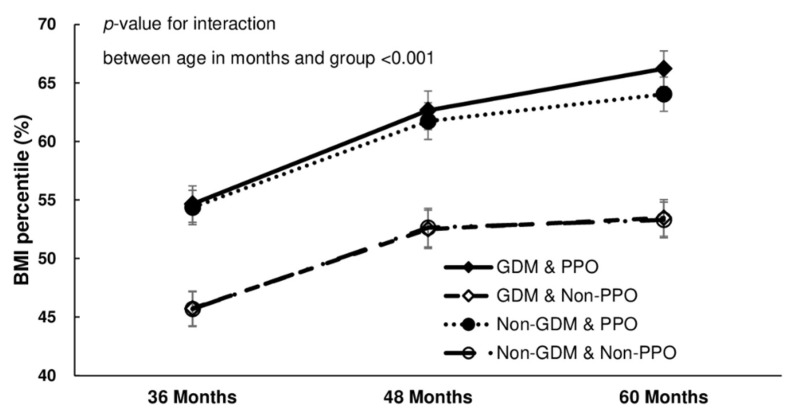
Body mass index percentile of the children according to age and group. BMI: body mass index, PPO: pre-pregnancy obesity, GDM: gestational diabetes mellitus.

A statistically significant difference was found in the prevalence of childhood obesity depending on the presence of PPO or GDM in mothers. Children whose mothers had both PPO and GDM had a significantly higher obesity prevalence and BMI percentile across all age groups (Table 2). Children at the age of 36 and 48 months had a higher BMI percentile if their mothers had PPO regardless of the presence of GDM, and the prevalence of obesity was the highest among children whose mothers had both. There was no difference in the average BMI percentile or obesity prevalence in children whose mothers did not have PPO. When the children were 60 months old, the groups were further subdivided. Particularly, the average BMI percentile was the highest in GDM with PPO, followed by non-GDM with PPO, and finally, GDM/non-GDM without PPO. The prevalence of obesity was lower in the non-GDM without PPO group than in the GDM without PPO group. The obesity prevalence did not differ significantly among the four groups depending on the age of the children, (interaction between age in months and groups, *p* = 0.658) as shown in Figure 2. However, there were statistically significant changes in the average BMI percentile among the groups (interaction between age in months and groups, *p* < 0.001). At the age of 60 months, children whose mothers had both PPO and GDM had a higher BMI compared to children with mothers belonging to other groups (Figure 3).

**Figure 4:**
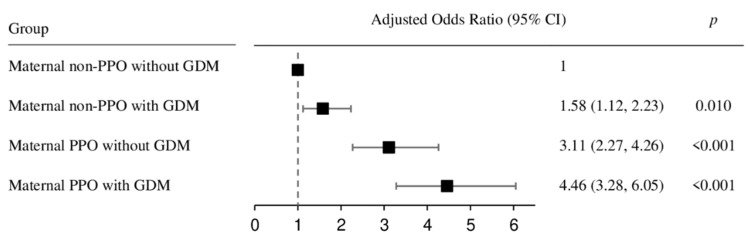
Adjusted odds ratio. CI: confidence interval, PPO: pre-pregnancy obesity, GDM: gestational diabetes mellitus.

Confounding variables in children, including birth weight, sex, breastfeeding, diet habits, TV or screen time, and exercise preference, were adjusted to examine the effects of maternal PPO and GDM on the development of childhood obesity at the age of 5 years. The results of this analysis are shown in Figure 4. Children whose mothers had both PPO and GDM had a 4.46 times higher risk (95% CI: 3.28–6.05, *p* < 0.001), and those whose mothers had only PPO had a 3.11 times higher risk (95% CI: 2.27–4.26, *p* < 0.001), and those whose mothers had only GDM had a 1.58 times higher risk (95% CI: 1.12–2.23, *p* = 0.010) of developing childhood obesity compared to children whose mothers had neither PPO nor GDM (reference group).

## 4. Discussion

This study compared the relative effects of maternal PPO and GDM on childhood obesity during early childhood. Our results showed that 5-year-old children who had mothers with GDM, PPO, or both GDM and PPO were at 1.58, 3.11, or 4.46 times higher risk, respectively, of developing childhood obesity than children whose mothers had neither GDM nor PPO. This finding confirmed that maternal PPO increases the risk of childhood obesity to a higher degree than maternal GDM, and the presence of both makes the risk even higher.

In this study, participant matching was accomplished depending on the presence of maternal PPO and GDM, rather than adjusting for PPO and then comparing the risk for childhood obesity. Although some previous studies have identified maternal GDM as a risk factor for childhood obesity, this finding did not remain significant after adjusting for PPO [26,27]. Meta-analyses have failed in drawing conclusions on the association between GDM and childhood obesity [19,28], and it is reported that maternal PPO should be considered to examine the association clearly [18]. However, the discussion on adjusting for maternal PPO remains controversial. Some studies claim that adjustment is necessary because if PPO is a confounding variable, it can falsely illustrate the association between GDM and childhood obesity [26,27]. In contrast, others argue that PPO and GDM are morbid expressions of identical conditions [14,29], and therefore, adjusting for PPO can reduce the effects of GDM, thus raising issues regarding over-adjustment [21]. Our study classified the participants into four groups and compared the risk of childhood obesity among these groups to disentangle the complex impact of maternal PPO and GDM. A similar study by Pirkola et al. [30] compared four groups of participants, who were categorized based on the combination of pre-pregnancy overweight and GDM, with a group without GDM risk factors to examine the risk of overweight among 16-year-old adolescents. Their results showed that pre-pregnancy overweight increased the risk for childhood overweight, and children whose mothers had both overweight and GDM had four times higher risk of being overweight [30]. The results are consistent with the findings of our study. By combining these findings, we interpret that, firstly, when mothers have both PPO and GDM, the risk for childhood obesity becomes higher than when mothers only have PPO or GDM, and secondly, this risk can have long-term effects, extending beyond early childhood.

Previous studies on maternal GDM have demonstrated the strong effect of PPO on childhood obesity [31,32,33,34]. In addition, the finding that maternal PPO without GDM can increase the risk for childhood obesity has been supported by consistent evidence [35,36,37]. It is suggested that developmental programming, genetic predisposition, and postnatal environmental factors are all involved in the mechanism of maternal PPO-induced childhood obesity [37]. Maternal obesity is a type of inflammatory state that increases insulin resistance and causes high blood glucose levels, resulting in intrauterine exposure to an over-nutritional environment in the fetus. This intrauterine mechanism explains the development of obesity among children whose mothers have GDM through changes in the developmental programming [38,39]; further, fetal overgrowth can start prior to the 20th week of gestation, that is, before the diagnosis of GDM [40]. Our study found that children whose mothers had PPO but were not diagnosed with GDM had a higher risk of developing obesity than those whose mothers were diagnosed with GDM. It can be presumed that this finding is due to children whose mothers have undiagnosed GDM and thus, are at an increased risk of undetected and untreated fetal hyperglycemia. Supporting this assumption, the study by Gomes et al. reported that the prevalence of obesity among 4-year-old children whose mothers had a negative oral glucose tolerance test and hemoglobin A1c ≥5.7% was greater than those whose mothers were diagnosed with GDM and were receiving treatment [41]. Boney et al. reported fetal hyperinsulinism when the mother was obese but not diagnosed with GDM [14], and Silverman et al. identified umbilical cord levels of C-peptide as a risk factor for obesity in 6-year-olds [31]. These findings can be explained by the assumption that children of obese mothers, who are exposed to mild maternal hyperglycemia, develop hyperinsulinism leading to childhood obesity through changes in developmental programming. In addition, it has been observed that children tend to have a significantly higher degree of insulin resistance when their mothers have both PPO and GDM, resulting in an increased risk of childhood obesity [42,43].

Our study examined the odds ratio of the prevalence of early childhood obesity based on the presence of maternal PPO and/or GDM. If mothers had PPO, children at the ages of 3, 4, and 5 years had a high prevalence of obesity, and this gradually increased with age. In particular, when the mother had both PPO and GDM, the risk for obesity was even higher in early childhood, and it increased dramatically with age. This finding is consistent with the results of previous studies on the association between maternal PPO and early childhood obesity [27,36,44,45]. In contrast to this, previous studies have reported inconsistent findings on the association between maternal GDM and childhood obesity in the early childhood period [27,32,33,37,46,47,48,49]. Some studies suggest that the risk for obesity increases with age [27,32,33], whereas others show no difference until children reach the age of 5 years [31,49]. Silverman et al. reported that the difference in childhood obesity at the age of 1 and 3 years was not significant, but with the second hit at the age of 4 years and sudden increase in weight at the age of 5 years, the differences in childhood obesity became significant [31]. They also showed that 50% of children were overweight at the age of 8 years [31]. Similarly, Baptiste-Roberts showed that although the difference in childhood obesity was not significant at the age of 3–4 years, it became significant at the age of 7 years [49]. In our study, when the children of mothers with GDM but without PPO were compared to the other groups, a significant difference was found when the children reached the age of 5 years, but there was no significant difference in obesity at the ages of 3 and 4 years. Considering the trend that the risk for obesity increases with age, more studies that include a follow-up period when the child reaches 5 years or older are necessary to confirm the association between GDM and the risk of childhood obesity.

There are some methodological advantages to this study. Firstly, to examine the existing inconsistent findings on the effects of PPO and GDM on childhood obesity, the study participants were categorized into four groups depending on the maternal presence of PPO and/or GDM, allowing researchers to evaluate both their individual and combined effects. Secondly, to eliminate the effect of birth year, maternal age, and income level, these variables were used to perform 1:1 matching and set a comparison group. In addition, the prevalence rate of childhood obesity was compared after adjusting for sex, birth weight, breastfeeding, and lifestyle factors, such as diet, TV or screen time, and exercise preference, to minimize the impact of confounding variables. Lastly, the participants were limited to children of mothers with GDM only, excluding mothers with pre-pregnancy diabetes, to eliminate the effects of different types of diabetes mellitus.

Among other strengths, this study examined the obesity risk in 3-, 4-, and 5-year-old children, who have been relatively less studied. Furthermore, the number of study participants used here was relatively large. In addition, this study verified the study participants with a universal screening tool used to assess GDM in all pregnant women from the NHIS that provides coverage to all citizens. Therefore, it carries a relatively low risk of bias or misclassification.

This study has a limitation pertaining to the examined variables because it used data from the NHID. Specifically, the presence of GDM was verified using diagnosis codes. As a result, information on blood glucose levels at the time of GDM diagnosis, treatment for GDM during pregnancy, blood glucose management during pregnancy, and increase in weight during pregnancy were not reflected. In addition, only mothers who gave birth at full-term to a single child were included in this study for appropriate comparisons across the groups. Therefore, results must be interpreted with careful thought.

## 5. Conclusions

This study identified and compared the risk of childhood obesity in early childhood by considering both maternal PPO and GDM. The results showed that maternal PPO conveys a higher risk for early childhood obesity up to the age of 5 years than maternal GDM and that the risk is even greater when mothers have both PPO and GDM. This study expands the understanding of the relative effects of these factors on early childhood obesity and confirms that maternal PPO and GDM increase the risk of childhood obesity, highlighting the need to manage PPO for the prevention of childhood obesity. As such, when a woman is obese before pregnancy and has GDM, her child is at very high risk for childhood obesity and should be considered a candidate for obesity prevention interventions. It is necessary to develop strategies for active obesity prevention in children before they reach the age of 5 years by either utilizing infant health screening appointments or providing separate interventions.

## Figures and Tables

**Table 1:** Baseline characteristics.

	GDM						Non-GDM						*p*-Value
	Maternal PPO (BMI ≥ 25)			Maternal Non-PPO (BMI < 25)			Maternal PPO (BMI ≥ 25)			Maternal Non-PPO (BMI < 25)			
**Total**	1319			1319			1319			1319			
**Maternal characteristics (Matched variables)**													
Age (years), mean (SD)	33.0	(4.0)		33.0	(4.0)		33.0	(4.0)		33.0	(4.0)		–
Income classification													–
High	190	(14.4)		190	(14.4)		190	(14.4)		190	(14.4)		
Middle	685	(51.9)		685	(51.9)		685	(51.9)		685	(51.9)		
Low	444	(33.7)		444	(33.7)		444	(33.7)		444	(33.7)		
BMI (kg/m^2^), mean (SD)	27.6	(2.5)	a	20.7	(2.1)	b	27.3	(2.2)	a	20.6	(2.0)	b	<0.001
**Children’s characteristics**													
Birth weight (kg), mean (SD)	3.30	(0.52)	a	3.20	(0.44)	b	3.29	(0.49)	a	3.19	(0.42)	b	<0.001
Sex (male)	667	(50.6)		698	(52.9)		683	(51.8)		699	(53.0)		0.576
Breastfeeding													0.087
Unknown	740	(56.1)		735	(55.7)		752	(57.0)		695	(52.7)		
Exclusive breastfeeding	226	(17.1)		242	(18.3)		236	(17.9)		261	(19.8)		
Mixed feeding	133	(10.1)		135	(10.2)		104	(7.9)		147	(11.1)		
Formula feeding	220	(16.7)		207	(15.7)		227	(17.2)		216	(16.4)		
Diet–sweetened drinks													0.444
High consumption	128	(9.7)		135	(10.3)		111	(8.5)		121	(9.2)		
Low consumption	1188	(90.3)		1181	(89.7)		1202	(91.5)		1197	(90.8)		
Diet–high consumption of fast food			a			b			b			b	<0.001
Yes	218	(16.5)		169	(12.8)		175	(13.3)		143	(10.8)		
No	1101	(83.7)		1150	(87.4)		1143	(87.1)		1176	(89.2)		
TV or screen time			a			b			c			b	<0.001
>2 h/day	518	(39.3)		410	(31.1)		464	(35.2)		379	(28.8)		
≤2 h/day	800	(60.7)		909	(68.9)		853	(64.8)		939	(71.2)		
Preference for exercise													0.471
Yes	983	(74.6)		953	(72.3)		952	(72.2)		963	(73.0)		
No	335	(25.4)		366	(27.7)		366	(27.8)		356	(27.0)		

The *p*-values are derived from the generalized estimating equation for clustered data; a, b, c: statistically significant results of the post-hoc analysis (*p* < 0.05); PPO: pre-pregnancy obesity, GDM: gestational diabetes mellitus, BMI: body mass index, SD: standard deviation.

**Table 2:** Distribution of BMI percentile and prevalence of obesity according to maternal GDM and PPO.

	Children in the GDM Group							Children in the Non-GDM Group						*p*-Value
	Maternal PPO (BMI ≥ 25)			Maternal Non-PPO (BMI < 25)				Maternal PPO (BMI ≥ 25)			Maternal Non-PPO (BMI < 25)			
**36 Months (30–36 Months)**														
N	1319			1319				1319			1319			
Mean (SD) of BMI percentile *	54.6	(28.8)	a	45.7	(27.4)	b		54.4	(27.2)	a	45.7	(27.5)	b	<0.001
Obesity **, *n* (%)	114	(8.6)	a	30	(2.3)	b		71	(5.4)	c	30	(2.3)	b	<0.001
**48 Months (42–48 Months)**														
N	1152			1133				1142			1140			
Mean (SD) of BMI percentile *	62.7	(28.6)	a	52.5	(28.1)	b		61.7	(27.1)	a	52.6	(28.4)	b	<0.001
Obesity **, *n* (%)	152	(13.2)	a	50	(4.4)	b		111	(9.7)	c	37	(3.2)	b	<0.001
**60 Months (54–60 Months)**														
N	1319			1319				1319			1319			
Mean (SD) of BMI percentile *	66.2	(28.2)	a	53.5	(29.1)		b	64.0	(27.1)	c	53.3	(28.2)	b	<0.001
Obesity **, *n* (%)	238	(18.0)	a	88	(6.7)		b	172	(13.0)	c	56	(4.2)	d	<0.001

* body mass index (BMI) percentile for age and sex, ** BMI percentile ≥ 95; *p*-value derived from the generalized estimating equation for clustered data; a, b, c, d: statistically significant results of the post-hoc analysis (*p* < 0.05); PPO: pre-pregnancy obesity; GDM: gestational diabetes mellitus; SD: standard deviation, BMI: body mass index.

## Data Availability

All available data generated or analyzed during this study are included in this article. The original research data are not available due to the strict confidentiality guidelines and regulations by the National Health Insurance Service. Access to research data is permitted only at the designated analysis center by the designated researcher, and data sharing is strictly prohibited.
